# Stimulatory Effect of Tofacitinib on Bone Marrow Adipocytes Differentiation

**DOI:** 10.3389/fendo.2022.881699

**Published:** 2022-07-06

**Authors:** Jean-Guillaume Letarouilly, Julien Paccou, Sammy Badr, Christophe Chauveau, Odile Broux, Aline Clabaut

**Affiliations:** ^1^ Université de Lille, Centre Hospitalier Universitaire CHU CENTRE HOSPITALIER UNIVERSITAIRE (CHU) Lille, MABLab ULR 4490, Service de Rhumatologie, Lille, France; ^2^ Université de Lille, Centre Hospitalier Universitaire (CHU) Lille, MABLab ULR 4490, Service de Radiologie et Imagerie Musculosquelettique, Lille, France; ^3^ Université Littoral Côte d’Opale, MABLab ULR 4490, Boulogne-sur-Mer, France

**Keywords:** tofacitinib, rheumatoid arthritis, human bone marrow-derived stromal cells, bone marrow adipocytes, osteoblasts, differentiation

## Abstract

**Background:**

Systemic inflammation is the main factor underlying secondary osteoporosis in patients with rheumatoid arthritis (RA). Janus kinase inhibitors (JAKi), such as tofacitinib (Tofa), can control systemic inflammation and may have beneficial effects on bone in various models. This might be due to direct effects on the bone microenvironment and not exclusively based on their anti-inflammatory function. Bone marrow adipocytes (BMAds) are abundant in the bone microenvironment. The effect of JAKi on BMAds is unknown, but evidence suggests that there is competition between human bone marrow-derived stromal cell (hBMSC) differentiation routes towards BMAds and osteoblasts (Ob) in osteoporosis.

**Objectives:**

The aims of the study are to determine whether Tofa influences BMAds and Ob derived from hBMSCs and to investigate the potential effects of Tofa on bone marrow adiposity in RA patients.

**Methods:**

To determine the effect of Tofa on cellular commitment, hBMSCs were differentiated to BMAds or OBs for 3 days together with Tofa at 200, 400, or 800 nM and TNFα. This study was also conducted using differentiated BMAds. The impact of Tofa was determined by gene and protein expression analysis and cell density monitoring. In parallel, in a pilot study of 9 RA patients treated with Tofa 5 mg twice a day (NCT04175886), the proton density fat fraction (PDFF) was measured using MRI at the lumbar spine at baseline and at 6 months.

**Results:**

In non-inflammatory conditions, the gene expression of Runx2 and Dlx5 decreased in Ob treated with Tofa (p <0.05). The gene expression of PPARγ2, C/EBPα, and Perilipin 1 were increased compared to controls (p <0.05) in BMAds treated with Tofa. Under inflammatory conditions, Tofa did not change the expression profiles of Ob compared to TNFα controls. In contrast, Tofa limited the negative effect of TNFα on BMAd differentiation (p <0.05). An increase in the density of differentiated BMAds treated with Tofa under TNFα was noted (p <0.001). These findings were consolidated by an increase in PDFF at 6 months of treatment with Tofa in RA patients (46.3 ± 7.0% versus 53.2 ± 9.2% p <0.01).

**Conclusion:**

Together, these results suggest a stimulatory effect of Tofa on BMAd commitment and differentiation, which does not support a positive effect of Tofa on bone.

## 1 Introduction

Rheumatoid arthritis (RA) is a systemic, immune-mediated disease characterized by synovitis and/or inflammation of periarticular structures and systemic inflammation with a prevalence in the range of 0.5–1.0% in Western countries (1, 2). Osteoporosis is a common feature in patients with RA and leads to an increased risk of fractures (3). In a meta-analysis, patients with RA had a significantly higher risk of fractures than patients without RA (relative risk  =  2.25, 95% CI [1.76–2.87]) (4).

Systemic inflammation is the main factor underlying this secondary osteoporosis in RA patients. Cytokines such as tumor necrosis factor alpha (TNFα) and interleukin 6 (IL-6) directly stimulate the maturation of osteoclasts and inhibit the differentiation of osteoblasts (Ob), leading to an imbalance in bone resorption and bone formation (5).

Twenty years ago, RA often led to joint destruction and considerable disability. Since then, scientific progress has prompted major advances in the treatment of the disease (2). Janus Kinase inhibitors (JAKi), which inhibit the JAK/STAT (signal transducer and activator of transcription) pathway can control the disease activity and thus prevent joint destruction (6–8). Moreover, JAKi, such as tofacitinib (Tofa), can control systemic inflammation and may have beneficial effects on bone. Tofa inhibits several STATs such as STAT1, STAT3, and STAT5 (9, 10). Two *in vitro* studies showed that Tofa may enhance osteogenic differentiation (11, 12). One study showed an increase in osteocalcin (OC) expression in murine bone marrow-derived stromal cells treated with Tofa 1 day after osteogenic induction. In contrast, the expression of runt-related transcription factor 2 (Runx2), the key transcription factor in osteogenesis, was not affected by Tofa (11). In the other study, Runx2 expression only increased under hypoxic conditions in human bone marrow-derived stromal cells (hBMSCs) treated with Tofa 7 days after osteogenic induction (12).

In a one-year pilot study, no changes in bone mineral density (BMD) by dual-energy X-ray absorptiometry (DXA) were observed in RA patients under Tofa (n = 26) whereas C-terminal collagen crosslinks (CTX) significantly decreased (13). Furthermore, Vidal et al. analyzed how treatment intervention with Tofa prevents the early disturbances of bone structure and mechanics in a rat model of adjuvant-induced arthritis (14). Tofa could control the inflammatory activity and, to increase cortical bone and trabecular hardness measured by nanoindentation, but did not reverse the effects of arthritis on the cortical and trabecular bone structure and on mechanical properties (14).

More studies are needed to understand the impact of Tofa on bone, as previous studies used different models and conditions.

This impact might be due to direct effects on the bone microenvironment and not exclusively based on its anti-inflammatory function. Bone marrow adipocytes (BMAds) are abundant in the bone microenvironment (15) and have been historically viewed as passive ‘fillers’ of bone marrow space that are metabolically inert (16). An increasing number of studies have shown that there is an association between decreased bone mass and the accumulation of bone marrow adipose tissue, suggesting a competition between hBMSCs differentiation routes toward BMAds and Ob in osteoporosis (15)

The study aimed to determine whether Tofa influences the cells of the bone microenvironment, such as BMAds and Ob derived from hBMSCs, under inflammatory and noninflammatory conditions. Then, in a prospective pilot study, we investigated the potential effects of Tofa on bone marrow adiposity and bone parameters in patients with RA.

## 2 Materials and Methods

### 2.1 Cell Culture Experiments

#### 2.1.1 Cell Culture and Induction of Osteogenic and Adipogenic Differentiation

Purified hBMSCs from 3 donors (1 female, 20 years; 2 males, 25 and 22 years) were purchased from RoosterBio (Frederick, MD, USA). Cells were specified as >90% positive for CD73, CD90, and CD105, and <10% negative for CD14, CD34, and CD45 surface marker expression.

Differentiation experiments were started when hBMSCs reached confluence (D0). To induce osteogenesis, hBMSCs were cultured in DMEM (Pan Biotech, Aidenbach, Germany) with 10% FCS (Pan Biotech, Aidenbach, Germany) supplemented with osteogenic inductors (50 μM ascorbic acid, 10 mM β-glycerophosphate, and 10^−8^ M vitamin D3 (Sigma-Aldrich)). For adipogenic differentiation, hBMSCs were cultured in DMEM with 10% FCS supplemented with adipogenic inductors (0.5 μM dexamethasone, 0.5 mM isobutyl-1methylxanthine, and 50 μM indomethacin (Sigma-Aldrich)). hBMSCs were cultured with 1 ng/ml TNFα (Gibco, Carlsbad, CA, USA) to mimic the inflammatory conditions in RA. The differentiation protocols were previously validated, as presented in **Supplementary Figure 1**.

**Figure 1:**
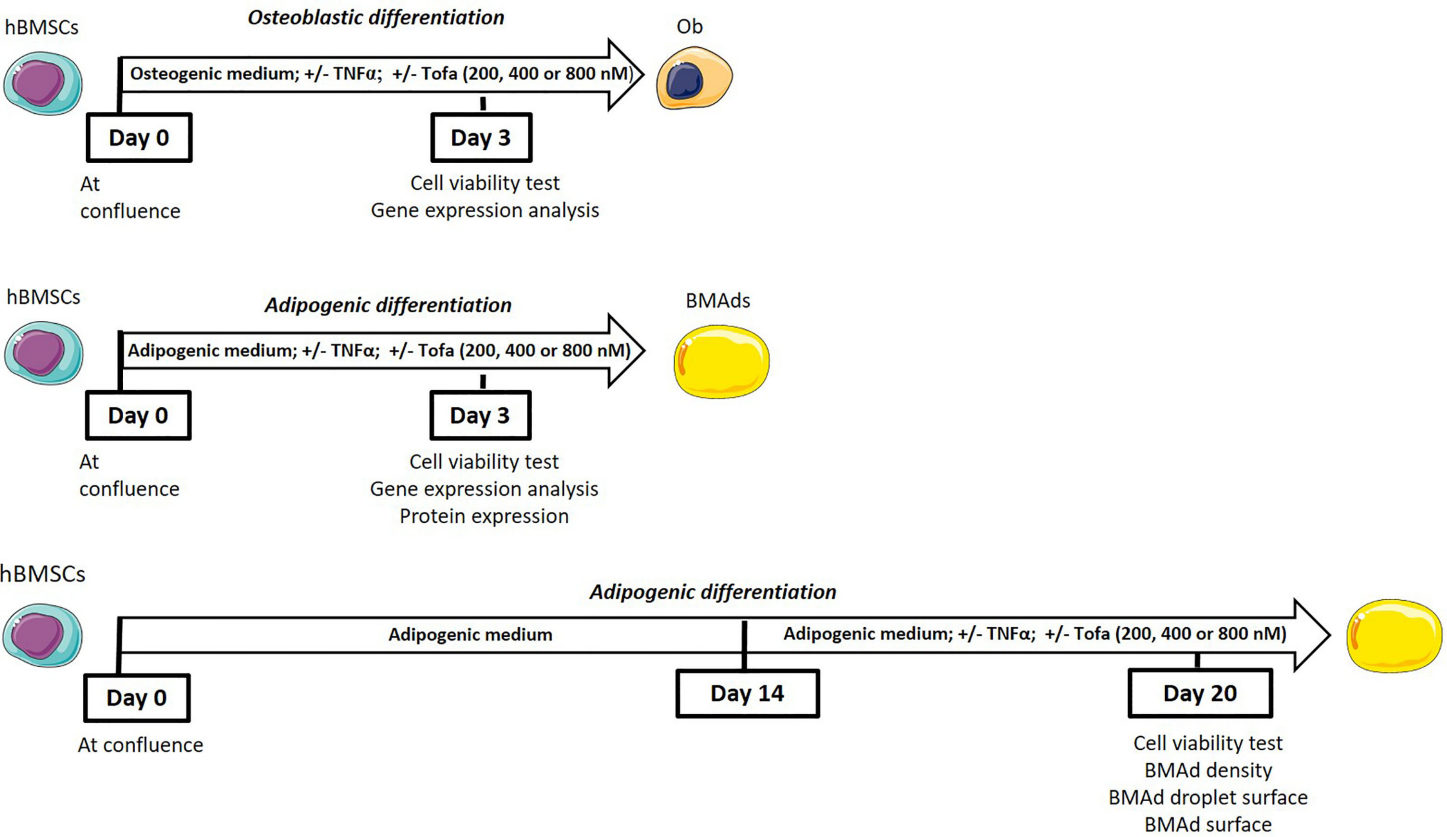
General scheme of the experiments.

To determine the impact of Tofa at an early commitment, hBMSCs were differentiated into Ob or BMAds in appropriate media for 3 days with TNFα and Tofa. To evaluate the effect of Tofa on fully differentiated BMAds, a similar treatment was applied for 6 days after 14 days of differentiation (**Figure 1**).

#### 2.1.2 Test Reagents

Tofa was graciously provided by Pfizer (New York, NY, USA), dissolved in sterile water and stored at −20 °C after sonication. Recombinant human TNFα was purchased from Gibco (Carlsbad, CA, USA), dissolved in sterile water and stored at −20 °C. Three concentrations of Tofa were used: 200, 400, and 800 nM. According to Pfizer, Tofa at 400 nM was considered as equivalent of the therapeutic dosage of Tofa used in RA (5 mg twice a day).

#### 2.1.3 Cell Viability Assay

In 96-well plates, Ob and BMAds were incubated with Tofa and TNF-α before being treated with 10 μl of WST-1 (Cell Proliferation Reagent WST-1; Roche, Mannheim, Germany). The mixture was then incubated for 4 h at 37 °C, after which the absorbance was measured at 450 nm. The reference wavelength was measured at 690 nm.

#### 2.1.4 Oil Red O Staining

After 20 days, differentiated BMAds were fixed in 2% paraformaldehyde for 15 min, washed in water, incubated with 60% isopropanol for 5 min, and stained with newly filtered Oil Red O solution for 10 min at room temperature. After staining, the cells were rinsed with water before counterstaining with hematoxylin solution, Gill no. 3 (Sigma Aldrich, St. Louis, MO, USA) for 5 min at room temperature.

#### 2.1.5 BMAd Density, BMAd Droplet Surface, and BMAd Surface

After 20 days, differentiated BMAds were fixed in 2% paraformaldehyde for 15 min, washed in water. BMAd density was assessed by counting the number of BMAds and the total number of cells in 12 images (6 per donor) for each condition. The BMAd droplet surface was assessed in 60 BMAds (30 per donor), and the BMAd surface was assessed in 100 BMAds (50 per donor) using ImageJ software (version 1.45).

#### 2.1.6 RNA Expression Measurement

##### 2.1.6.1 RNA Isolation

Total RNA was extracted from Ob and BMAd using an RNeasy^®^ Micro Kit including a DNase I digestion step (Qiagen, Hilden, Germany), according to the instructions of the manufacturer, and quantified by Nanodrop at 260 nm wavelength.

##### 2.1.6.2 mRNA Expression Analysis

Total RNA was reverse transcribed using a Maxima First-Strand cDNA Synthesis kit (Thermo Fisher Scientific, Waltham, MA, USA) and subjected to quantitative real-time PCR on the StepOnePlus^®^ system (Applied Biosystems, Foster City, CA, USA) using POWER SYBR^®^ Green PCR Master Mix (Thermo Fisher Scientific, Waltham, MA USA) and specific primers designed using Oligo 6 software (MedProbe), or Taqman Universal Master Mix II (Applied Biosystems, Foster City, CA, USA) and specific probes (**Table 1**). Relative gene expression levels were normalized to YWHAZ (tyrosine 3-monooxygenase/tryptophan 5-monooxygenase activation protein) and PPIA (peptidylprolyl isomerase A) for Ob and RNAPol2 (RNA polymerase II) and YWAZ transcripts for BMAds and determined using the 2 ^−ΔΔCt^ method.

**Table 1:** Primer sequences and conditions of PCR.

cDNA	GenBank	Forward and reverse primers	Ta (°C)	Product (pb)
*RNAPol2*	NM_000937	F: 5’-CCAAGCAGGACGTAATAGAGG-3’ R: 5’-GCGGTCTAGCAACTCGTTCT-3’	55	193
*PPIA*	NM_021130	F: 5’-ACCGTGTTCTTCGACATTGC-3’ R: 5’-CAGGACCCGTATGCTTTAGGA-3’	55	274
*PPARγ2*	NM_015869	F: 5’-CAAACCCCTATTCCATGCTGTT-3’ R: 5’-AATGGCATCTCTGTGTCAACC-3’	53	135
*C/EBPα*	NM_004364	F: 5’-ACTGGGACCCTCAGCCTTG-3’ R: 5’-TGGACTGATCGTGCTTCGTG-3’	55	75
*IL6*	NM_000600	F: 5’-CAATGAGGAGACTTGCCTGG-3’ R: 5’-GCACAGCTCTGGCTTGTTCC-3’	53	113
*PLIN 1*	NM_002666	F: 5’-GAGACACTGCGGAATTTGC-3’ R: 5’-ATCGAGAGAGGGTGTTGGTC-3’	58,2	222
*Ywhaz*	NM_145690	F: 5’-GGTCATCTTGGAGGGTCGTC-3’ R: 5’-GTCATCACCAGCGGCAAC-3’	55	245
*Runx2*	AF001450	F: 5’-GCTGTTATGAAAAACCAAGT-3’ R: 5’-GGGAGGATTTGTGAAGAC-3’	60	108
*OC*	NM_199173	F: 5’-ATGAGAGCCCTCACACTCCTC-3’ R: 5’-GCCGTAGAAGCGCCGATAGGC-3’	55	293
*Dlx5*	BC006226	F: 5’-CCAGAGAAAGAAGTGACCGA-3’ R: 5’-CCTGTGTTTGTGTCAATCCC-3’	55	190

The primer sequences, annealing temperatures (Ta), lengths of the corresponding PCR products and GenBank accession numbers are shown. F, Forward; R, Reverse. IL6, Interleukin 6; PLIN 1, Perilipin 1; C/EBPα, CCAAT/enhancer-binding protein alpha; PPARγ2, Peroxisome proliferator-activated receptor gamma 2; PPIA, peptidylpropyl isomerase; Runx2, Runt-related transcription factor 2; Ywhaz, Tyrosine 3-monooxygenase/tryptophan 5-monooxygenase activation protein; OC, Osteocalcin; Dlx5, Distal-less homeobox 5.

#### 2.1.7 Protein Sampling, Quantification, and Western Blot Analysis

For sampling, BMAds were washed in ice-cold PBS (Pan Biotech, Aidenbach, Germany) and lysed in lysis buffer [HEPES, MgCl2, Glycerol, KCl, EDTA, DTT] supplemented with Halt Protease Inhibitor Single-Use Cocktail (Thermo Fisher Scientific, Waltham, MA, USA) and sodium orthovanadate. Cellular debris was discarded by centrifugation after cell lysis. To adjust the protein concentration, extracts were quantified using the DC Protein Assay Kit (Bio-Rad, Hercules, CA, USA) according to the instructions of the manufacturer and denatured in 4× Laemmli Sample Buffer (Bio-Rad, Hercules, CA, USA). Protein extracts were separated on 8% SDS–polyacrylamide gels and transferred onto 0.2 µm nitrocellulose membranes (Bio-Rad, Hercules, CA, USA) using a Trans-Blot Turbo System (semidry transfer) (Bio-Rad, Hercules, CA, USA). The membranes were blocked in 5% bovine serum albumin (Sigma Aldrich, St. Louis, MO, USA) for phosphorylated proteins or with 5% skimmed milk for the other proteins in Tris-buffered saline solution with 0.05% Tween 20 (Acros Organics, Morris Plains, NJ, USA) for 1 h. Blots were probed with antibodies against the following targets overnight: anti-phospho-STAT3 (Tyr705; Cell Signaling Technology, Danvers, MA, USA), anti-STAT 3 (D3Z2G; Cell Signaling Technology, Danvers, MA, USA), anti-Actin (A2066, Sigma Aldrich, St. Louis, MO, USA) and anti-Perilipin (D1D8; Cell Signaling Technology, Danvers, MA, USA). Horseradish peroxidase–conjugated immunoglobulin G (Santa Cruz Biotechnology, Dallas, TX, USA) was used as secondary antibody. Blots were developed using Amersham ECL Prime Western Blotting Detection Reagent substrate (GE Healthcare, Chicago, IL, USA) in a chemiluminescence imager (Amersham Imager 600, GE Healthcare, Chicago, IL, USA). Band intensities were measured using ImageQuant TL (GE Healthcare, Chicago, IL, USA). Band intensity was semiquantified using ImageJ software (version 1.45). Actin was used as an internal control for normalization.

### 2.2 Clinical Research

#### 2.2.1 Study Design and Patients

The TOFAT study was an open, prospective 6-month follow-up study (NCT04175886) assessing the impact of Tofa on bone marrow adiposity, BMD, and body composition in RA patients 18 years and older. Key inclusion criteria include 2010 RA ACR/EULAR classification criteria (17), and an indication for Tofa. Key exclusion criteria included oral corticosteroids >10 mg prednisone/day; patients on or considering a restrictive diet during the study period; patients undertaking or planning to undertake an intense exercise program; history of treatment with bone active substances such as bisphosphonates; weight >160 kg; and any MRI contraindication. In patients who were receiving Biological Disease-Modifying Antirheumatic Drugs (bDMARDs), a five-half-life washout period was required between bDMARD interruption and inclusion in the study.

#### 2.2.2 Study Approval

The study protocol was approved by the local Institutional Review Board (2019–001159–37), and the study procedures complied with the ethical standards of the relevant institutional and national Human Experimentation Ethics Committees (reference CPP 40/19). All patients provided written informed consent. The study was designed to include 38 patients but was prematurely stopped due to the results of the safety Phase IV randomized clinical trial (18). Only 10 patients were included, and one participant was excluded at follow-up due to discontinuation of Tofa before 6 months.

#### 2.2.3 Clinical Assessment

Demographic and clinical characteristics were recorded, particularly the 28-joint Disease Activity Score (DAS28), adjusted for C Reactive Protein (CRP) levels, current use of conventional synthetic disease-modifying antirheumatic drugs (csDMARDs) and corticosteroids, and physical activity using the International Physical Activity Questionnaire-Short Form (IPAQ-SF) and the handgrip test. Past use of bDMARDs was also recorded.

#### 2.2.4 Bone Mineral Density and Body Composition Measurements Using DXA Scan

Bone mineral density (BMD) in RA patients was measured at the lumbar spine (L1–L4) and at the nondominant hip using a DXA scan (HOLOGIC Horizon W S/N 300869M).

All RA patients underwent total body DXA scanning (HOLOGIC Horizon W S/N 300869M). Fat, lean, and bone mass for the total body and per region (arms, legs, and trunk) were measured and analyzed using validated software from the manufacturer (version 13.6.0.5). Body fat percentage (BFP, %) was calculated as the proportion of total fat mass (TFM) to total mass. Appendicular lean mass (kg) was computed as the sum of the tissue compartment (lean) of both arms and legs. Visceral adipose tissue (VAT, cm²) was recorded.

#### 2.2.5 Laboratory Variables

Fasting (at least 8 h) blood samples were obtained. Serum cross-laps (CTX) were measured by a chemiluminescence assay using the IDS-iSYS Multi-Discipline Automated Analyzer (Immunodiagnostic Systems, Inc., Fountain Hills, AZ). Plasma concentrations of leptin were measured by ELISA using the E07 kit provided by Mediagnost (Reutlinger, Germany).

#### 2.2.6 Bone Marrow Adiposity Measurement by MRI

##### 2.2.6.1 Imaging Acquisition

All subjects underwent an MRI examination on a 3 Tesla system (Ingenia; Philips Healthcare, Best, Netherlands) using the built-in 12-channel posterior body coil and a 16-channel anterior coil, under the supervision of a senior musculoskeletal radiologist. Patients were positioned head-first in the supine position. A conventional imaging protocol was performed first, including T1- and T2-weighted 2-point Dixon turbo-spin echo (TSE) acquisitions in the sagittal plane, followed by an optional T2-weighted acquisition in the axial plane based on clinical history and the decision of the radiologist.

Immediately after this clinical exploration, bone marrow adiposity quantification (proton density fat fraction, PDFF) was achieved using a six-echo three-dimensional gradient echo sequence (mDixon-Quant; Philips Healthcare, Best, Netherlands), permitting a chemical shift encoding-based water-fat separation at the lumbar spine (sagittal). Imaging parameters were repetition time (TR)/echo time (TE)/ΔTE: 11/1.43/1.1 ms; field of view (FOV): 220 × 220 mm; voxel size: 1.8 × 1.8 mm; slice thickness: 3 mm; number of excitations: 1; no SENSE acceleration; fold-over direction: foot-head; bandwidth: 1,563 Hz and scan time: 1 min 41 s. In both situations, a low flip angle of 3° was used to minimize T1-bias (19).

##### 2.2.6.2 Bone Marrow Adiposity Quantification

A senior musculoskeletal radiologist (SB) reviewed all examinations on a dedicated workstation (IntelliSpace Porta; Phillips Healthcare, Best, the Netherlands). A systematic morphological assessment was conducted first, looking for lumbosacral transitional vertebrae, degenerative changes, and bone marrow replacement lesions. Based on the 3 most sagittal slices acquired using the mDixon-Quant sequence, polygonal regions of interest (polygonal ROI) were drawn in the vertebral body of L1 to L4, avoiding subchondral bone, replacing lesions, severe degenerative changes, and the basivertebral vein. For each subject, a mean proton density fat fraction (PDFF) was calculated as an average of all measured values in the lumbar spine, from L1 to L4.

### 2.3 Statistical Analysis

Categorical variables are expressed as numbers (percentage), and continuous variables are expressed as the means (standard deviation, SD, or standard error of the mean, SEM). The normality of model residuals was assessed graphically and using a D’Agostino’s K-squared test. Changes were examined during treatment by comparing baseline and 6-month values using a paired t-test or a Wilcoxon signed-rank test, depending on the normality of intra-patient differences. In the *in vitro* study, all experiments were repeated at least three times, and the Mann–Whitney U test and Student’s t-test were used according to the distribution of the data. Statistical testing was performed at the two-tailed α level of 0.05. Data were analyzed using the GraphPad Prism software package, release 5.04 (GraphPad Software, San Diego, CA, USA).

## 3 Results

### 3.1 Tofa Does Not Inhibit Survival of hBMSCs During Ob or BMAd Differentiation

**Figure 2:**
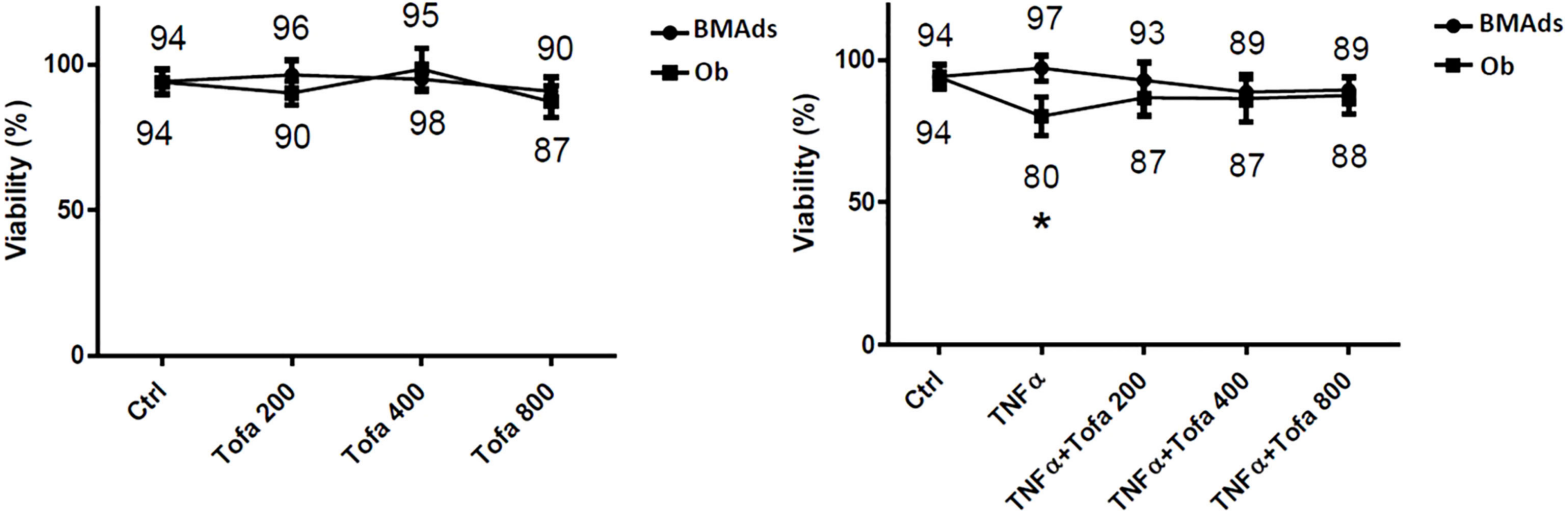
Effect of Tofa and TNFα on the cellular viability of hBMSCs. Ob and BMAds were differentiated from hBMSCs for 3 days and treated with Tofa (200, 400, and 800 nM) and TNFα (1 ng/ml). Cell viability was assessed using the WST-1 method, and the percentage of viability is shown above and below the symbols. Data represent the mean ± SD of four independent experiments. ∗p <0.05 versus control (untreated group) by Mann–Whitney U test.

Cytotoxicity tests were performed to assess whether cell survival is influenced by Tofa concentrations at 200, 400, and 800 nM (**Figure 2**).

We observed no changes during BMAd differentiation (BMAds at 3 days) regarding proinflammatory or noninflammatory conditions or the doses of Tofa tested. In Ob differentiated at 3 days, Tofa did not impact cellular viability but rather seemed to improve it when the cells were in the presence of TNFα. In fact, the significant decrease in cellular viability observed in Ob TNFα compared to the control was not observed in Ob TNFα + Tofa.

### 3.2 Tofa Treatment Has Little Effect on Ob Differentiation But Increases BMAds Differentiation Under Noninflammatory Conditions

To determine the impact of Tofa on cellular commitment, gene expression analysis was performed on specific markers related to Ob and BMAds differentiation. The results of quantitative real-time RT-PCR are displayed as the relative expression of mRNA levels in the Tofa-treated group compared to the control (untreated group).

**Figure 3:**
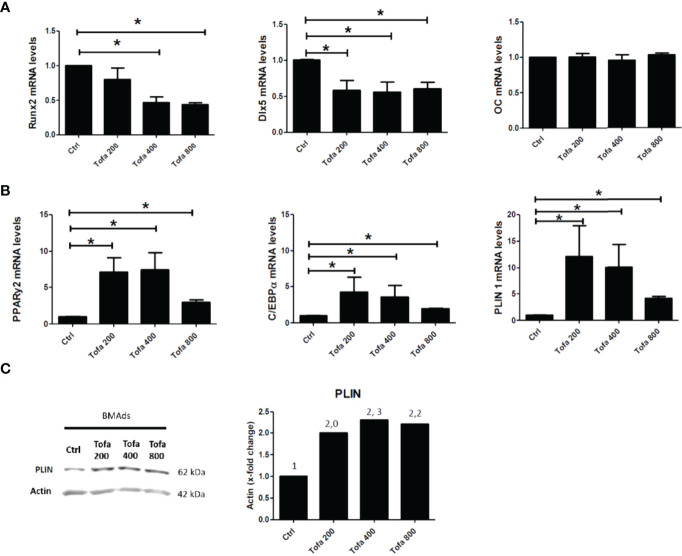
Effect of Tofa on Ob and BMAds differentiation under noninflammatory conditions. Ob and BMAds were differentiated from hBMSCs and treated with Tofa (200, 400, and 800 nM) for 3 days. Ctrl: Ob or BMAds untreated **(A, B)** Quantitative RT-PCR analysis of osteoblast **(A)** and adipocyte-specific genes on day 3 **(B)**. Data are expressed as means ± SEM. Statistical significance was calculated using the Mann–Whitney U test (*p <0.05). **(C)** Western blot analysis of hBMSCs cultivated 9 days in adipogenic medium with Tofa 200, 400, and 800 nM. Representative western blots for PLIN with actin as a loading control and corresponding quantification of protein quantity (PLIN/Actin).

Under noninflammatory conditions, we showed that Tofa significantly decreased expression levels of Runx2 at 400 nM and at 800 nM and Dlx5 (Distal-less homeobox 5, another key transcription factor of Ob differentiation) for all concentrations compared with the Ob control (p <0.05) (**Figure 3A**). No effect of Tofa on osteocalcin gene expression levels was observed (**Figure 3A**).

Conversely, BMAds treated with Tofa exhibited a significant increase in the gene expression of PPARγ2, C/EBPα (two key transcription factors of BMAds differentiation) and Perilipin 1 (PLIN 1), the most specific marker associated with lipid droplet) compared with BMAds controls (p <0.05). An increase was observed from 200-nM Tofa for each marker (**Figure 3B**).

The increase in expression of PLIN was also confirmed at the protein level in BMAds cultivated for 9 days in adipogenic medium, whatever the Tofa concentration applied (**Figure 3C**).

### 3.3 Tofa Limits the Negative Effect of TNFα During Differentiation But Only in an Adipocytic Context

**Figure 4:**
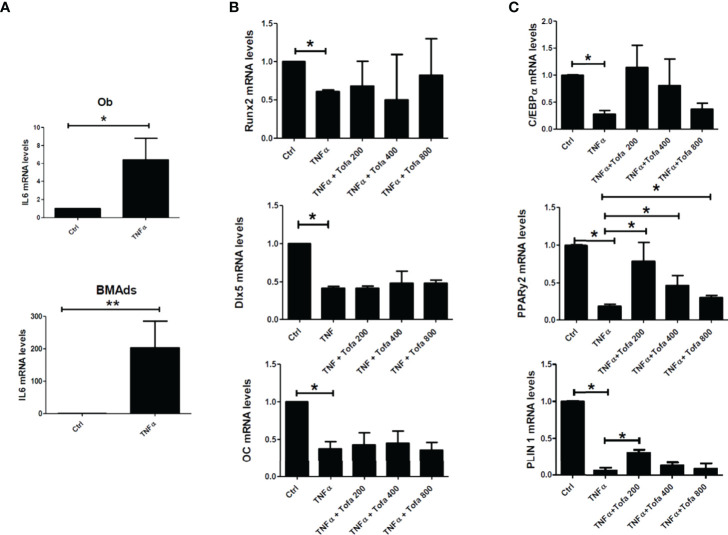
Effect of Tofa on Ob and BMAd differentiation under inflammatory conditions. Ob and BMAds were differentiated from hBMSCs and treated with Tofa (200, 400, and 800 nM) and TNFα (1 ng/ml) for 3 days. Ctrl: Ob or BMAds untreated **(A–C)** Quantitative RT-PCR analysis of IL-6 **(A)**, osteoblast **(B)**, and adipocyte-specific genes on day 3 **(C)**. Data are expressed as the means ± SEM. Statistical significance was calculated using the Mann–Whitney U test (*p <0.05, **p <0.01).

As expected, the addition of TNFα stimulated proinflammatory cytokine expression in both cultures, as demonstrated here by the increase in IL6 gene expression up to 300-fold higher in BMAds TNFα and 9-fold in Ob TNFα compared to the controls, which were untreated BMAds and Ob (**Figure 4A**).

TNFα also impacted Ob and BMAd differentiation, marked by a significant decrease in Runx2 (40%), Dlx5 (58%), OC (62%) (**Figure 4B**), PPARy2 (83.5%), C/EBPα (80%), and PLIN 1 (97%) (**Figure 4C**).

The addition of Tofa to TNFα did not change the expression profiles of Ob compared to TNFα controls (**Figure 4B**). In contrast, analysis of PPARy2 gene expression revealed that the addition of Tofa limited the negative effect of TNFα on BMAd differentiation (**Figure 4C**). Expression levels of PPARy2 were increased by an average of 4, 2.5, and 1.3 times, in response to Tofa 200, 400, and 800 nM, compared to TNFα-treated cells.

### 3.4 A Positive Effect of Tofa on BMAds Under Inflammatory Condition Was Also Observed at a Later Stage of Maturation

**Figure 5:**
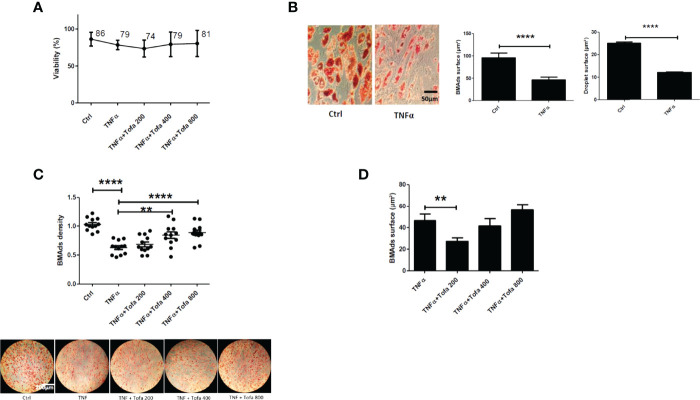
Effect of Tofa on differentiated BMAds under inflammatory condition. BMAds were differentiated from hBMSCs for 14 days and then cultured in the presence or absence of Tofa and TNFα for 6 days. **(A)** Cell viability was assessed using the WST-1 method, and the percentage of viability is shown above and below the symbols. Data represent the mean ± SD of four independent experiments. **(B)** Representative image showing the effect of TNFα treatment on adipogenesis, visualized by the uptake of oil red into lipid vacuoles (red). Quantification of BMAd surface and droplet surface (µm^2^) after TNFα treatment. **(C)** Quantification of the number of BMAds (number of BMAds/number of total cells) and **(D)** BMAd surface, after TNFα and Tofa treatment. **(B–D)** Data are expressed as the means ± SEM (n = 2). Statistical significance was calculated using Mann–Whitney U test for non-parametric variables and Student’s t-test (**p <0.01, ****p <0.0001).

Given our results above, we chose to further evaluate the action of Tofa on BMAds after 14 days of differentiation followed by 6 days of TNFα + Tofa treatment. Previously, the effect of TNFα + Tofa on the cellular viability of differentiated BMAds was assessed and no change was observed compared to the control, BMAds + TNFα (**Figure 5A**).

To evaluate the effect of Tofa, optical images of BMAds treated with TNFα + Tofa at 200, 400, and 800 nM were analyzed and BMAd density was determined (number of BMAds/number of total cells). First, we observed a phenotype change and a decrease in BMAd density in response to TNFα treatment. In fact, TNFα induced a phenotypic conversion of BMAds toward a fibroblastic phenotype with a drastic decrease in BMAd surface (p <0.001) and droplet surface compared to controls (p <0.001) (**Figure 5B**).

The addition of Tofa to the culture media limited the phenotypic conversion and maintained a BMAd density close to that of the control (untreated cells) with a dose–response effect (**Figure 5C**). The surface of BMAds was affected at 200 nM (p <0.01), but not at 400 or 800-nM Tofa (**Figure 5D**).

### 3.5 Tofa Inhibits pSTAT3 in BMAds

**Figure 6:**
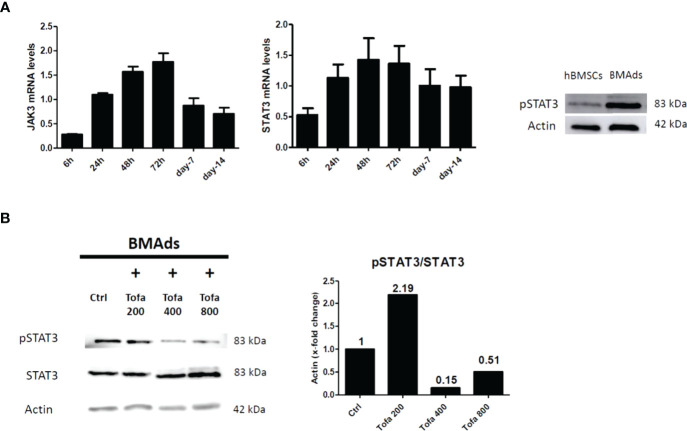
Analysis of pSTAT3 profile in BMAds. Quantitative RT-PCR analysis of JAK3 and STAT3 **(A)** during adipocyte differentiation compared to day 0 (hBMSCs confluents). STAT3 activity (pSTAT3) during adipocyte differentiation was demonstrated by western blot analysis. BMAds were differentiated 48 h in adipogenic medium and compared to hBMSCs. **(B)** Western blot analysis was used to determine relative pSTAT3/STAT3 expression in BMAds after 48 h of differentiation and treated 3 days with Tofa (200, 400, and 800 nM). Actin was used as a loading control. Data are representative of three independent experiments.

Although TOFA is a specific inhibitor of JAK1/3 and inhibits STAT1/3 phosphorylation, we were preferentially interested in the phosphorylation profile of STAT3 (pSTAT3) after 3 days of hBMSC differentiation. Indeed, the JAK3/STAT3 signaling pathway was upregulated during adipocyte differentiation and associated with an upregulation of pSTAT3 expression profile (**Figure 6A**). For JAK1/STAT1, the expression levels were stable during differentiation and were similar to those found in hBMSCs (data not shown). In response to Tofa, pSTAT3 markedly decreased in BMAds compared to controls (untreated cells), suggesting a potential role for STAT3 in the expansion of bone marrow adipose tissue (**Figure 6B**).

### 3.6 Bone Marrow Adiposity is Increased in RA Patients Treated With Tofa

#### 3.6.1 Baseline Characteristics of Patients

Age and disease duration (mean (SD)) were 58.0 (10.1) years and 7.3 (8.3) years, respectively. DAS28-CRP was 3.81 (1.2). Five patients were currently receiving corticosteroids (prednisone) at a dosage of less than 10 mg per day (6.8 (2.9) mg per day). Two patients had previously received at least one anti-TNFα treatment.

#### 3.6.2 Clinical, Physical and Body Composition Changes in RA Patients During Treatment With Tofa

One participant was excluded at follow-up due to discontinuation of Tofa before 6 months. No significant change in disease activity was noted (DAS 28 CRP: 3.81 (1.19) vs 3.00 (1.06), p = 0.16). After 6 months of Tofa, a significant increase in the handgrip test was noted, whereas no change in BMI was observed (**Table 2**).

**Table 2:** Changes in rheumatoid arthritis patients treated with Tofacitinib for 6 months.

Patients	M0N = 9	M6N = 9	Absolute difference	P-value
Body mass index, kg/m²	25.9 (3.4)	26.1 (6.3)	+0.2	0.64
Handgrip test, kg	**22.5 (8.9)**	**27.9 (14.0)**	**+5.4**	**0.01**
IPAQ-SF, MET-min/week	6,203 (8,461)	7,110 (4,111)	+907	0.49
Body Fat Percentage, %	3939.4 (8.5)	39.4 (9.0)	0	0.46
Appendicular lean mass, kg	16.6 (4.5)	16.7 (4.9)	+0.1	0.50
Visceral Adipose Tissue, cm²	150 (81)	173 (88)	+23	0.18
Lumbar spine BMD, g/cm²	1.011 (0.142)	0.996 (0.146)	−0.015	0.058
Femoral neck BMD, g/cm²	0.728 (0.108)	0.713 (0.095)	−0.015	0.30
Total hip BMD, g/cm²	0.902 (0.130)	0.891 (0.105)	−0.011	0.82
Lumbar spine PDFF (%)	**46.3 (7.0)**	**53.2 (9.2)**	**+6.9**	**0.008**
CTX, pmol/L	3,503 (1,830)	2,712 (2,196)	−791	0.91
Leptine, ng/L	14.9 (19.7)	19.4 (24.9)	+4.5	0.30

Data reported as mean (Standard Deviation) unless otherwise indicated. CTX , Cross laps; DAS, Disease Activity Score; IPAQ-SF, International Physical Activity Questionnaire—Short Form; MET, Metabolic Equivalent of Task; PDFF, Proton Density Fat Fraction. Bold values mean statistically significant.

**Figure 7:**
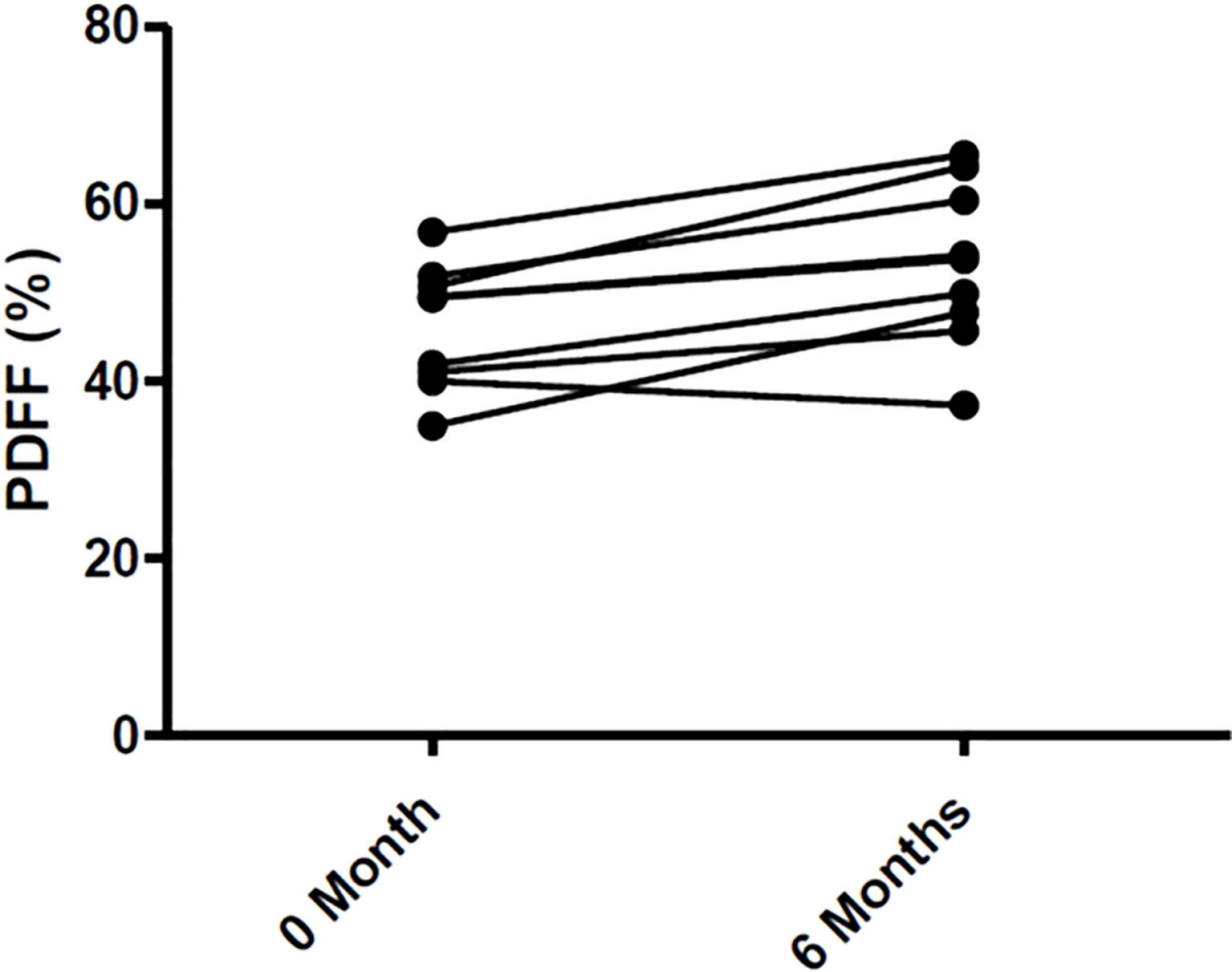
Changes of Bone Marrow Fat in 9 RA patients treated with Tofa. Bone marrow fat was assessed by MRI at lumbar spine in the 9 patients treated with Tofa during 6 months. PDFF, Proton Density Fat Fraction.

Changes in body composition, BMD, and lumbar spine PDFF are presented in **Table 2**. After 6 months of Tofa, a significant increase in the lumbar spine PDFF was noted (46.3% (7.0) vs 53.2% (9.2), p = 0.008) (**Figure 7**), whereas no changes in BMD or body composition were observed.

No significant changes in CTX (3,503 (1,830) pmol/L vs. 2,712 (2,196) pmol/L, p = 0.91), or leptin (14.9 (19.7) ng/L vs. 19.4 (24.9) ng/L, p = 0.30) levels were observed at 6 months.

## 4 Discussion

Studies have reported an increased risk of bone loss and fracture in RA patients (20). Tofa may have beneficial effects on bone, which might be due to direct effects on the bone microenvironment and not exclusively based on its anti-inflammatory function. As common progenitor cells of BMAds and Ob, hBMSCs are delicately balanced in their differentiation commitment (21, 22). In osteoporosis, numerous *in vitro* and clinical investigations have demonstrated the ability of hBMSCs to differentiate in a preferential way toward BMAds (23–28). In this study, the ability of Tofa to counteract this imbalance was investigated. For that, the effects of Tofa on the viability and Ob and BMAd differentiation of hBMSCs were assessed.

First, the lack of data on this subject led us to explore the influence of Tofa under noninflammatory conditions. The results at the cellular and molecular levels revealed that Tofa promoted adipocyte differentiation, even at low concentrations, but decreased the Ob differentiation after 3 days of treatment. Tofa has a fast onset of action, as the impact of Tofa on adipocytes and Ob differentiation has already been observed after only 3 days of treatment.

Concomitantly to our work, a stimulating effect of Tofa on differentiated adipocytes has also been reported on primary human bone marrow cells isolated from patients undergoing orthopedic surgery (29). In this study, Russell et al. reported an increase in the number of adipocytes for cells differentiated and treated by Tofa for 14 or 21 days.

Considering those results, we investigated the origin of the expansion of the adipose tissue treated with Tofa. Adipose tissue expansion results either from an increase in the size of differentiated adipocytes (adipocyte hypertrophy) and/or an increase in the formation of new adipocytes (adipocyte hyperplasia). This question was also raised by Russell et al. and was answered through the experiments conducted in our study. Indeed, measurements of the BMAd surface did not show an increase in the surface under Tofa, but the number of adipocytes was increased for the highest concentrations of Tofa (400 and 800 nM). These new findings confirm that Tofa participates in the expansion of adipose tissue by recruiting more preadipocytes and activating their differentiation. Consequently, we can also conclude that the increase in PLIN protein expression observed by western-blotting is therefore related to the appearance of new adipocytes rather than to the increase in the number of lipid droplets in the adipocytes already formed. This impact could be mediated through STAT3 inactivation. However, it has been shown in different studies that STAT3 is a rather pro-adipogenic factor (29, 30). Furthermore, in another *in vitro* model, Tofa treatment also resulted in off-target activation, leading to great complexity in its signaling (31, 32). Therefore, further studies are therefore needed to understand the mechanism that induces increased adipogenesis under Tofa.

Second, to get as close as possible to the inflammatory conditions of pathology, we treated the cultures with TNFα in addition to Tofa. Numerous studies have focused on the effect of TNFα on Ob and have shown the deleterious effect of this molecule on differentiation, characterized by a drastic decrease in Ob factors such as Runx2 or Dlx5 (33). However, even when gene expression levels of Runx2/Dlx5 were lowered by the effect of TNFα, tofacitinib was unable to counteract the action of TNFα and to stimulate Runx2 or Dlx5 as in noninflammatory conditions. These results support the latest published results and oppose the osteoanabolic effect previously described in the literature (11, 12). Russell et al. observed that Tofa did not affect the Ob differentiation at 14 days, as assessed by ALP activity and accumulation of calcium (34). Similarly, Gaber’s experiments did not reveal any changes in the gene expression of osteoblastic markers in hBMSCs treated with Tofa 7 days after osteogenic induction, grown under standard conditions. An increase in Runx2 expression was observed only under hypoxic conditions (12).

In contrast, Tofa appears to attenuate the deleterious effect of TNFα on the cellular viability of hBMSCs placed in osteogenic medium. In a recent study and in a different context, the protective effect of Tofa on cellular viability has also been reported (35). The authors used an oxygen-glucose deprivation/reoxygenation (OGD/R)-induced normal rat small intestinal epithelial cell model to simulate the physiological environment of intestinal I/R injury, treated or not with Tofa. The results showed that Tofa exerted protective effects on oxidative stress and inflammation in these cells but also on apoptosis during OGD/R. This effect could be mediated through the inhibition of the JAK/STAT3 pathway since the use of an agonist of this pathway partially abrogated the beneficial effect of Tofa.

In humans, the effects of Tofa on bone marrow adiposity in RA patients have never been evaluated. We have now conducted a prospective pilot study in 9 patients with RA, in which Tofa increased lumbar spine PDFF and resulted in no changes in BMD or body composition over 6 months. These clinical findings reinforce the *in vitro* results on the stimulatory effect of Tofa on BMAd differentiation, even if several limitations are to be underlined in this study. The hBMSCs used were obtained from healthy donors and treated with TNFα, which only partially reflected the pathology. It would be interesting to validate these results using hBMSCs collected from RA patients, especially since the clinical data obtained predicts a similar positive effect of Tofa on adipocyte differentiation. Clinically, we acknowledge that 6 months of follow-up is a short period to evaluate the BMD and body composition in patients treated with Tofa, and that 9 patients is a small population, but despite that, we were able to observe an increase in lumbar spine PDFF.

In conclusion, *in vitro* and clinical results suggest a stimulatory effect of Tofa on BMAd commitment and differentiation, which does not seem to support the beneficial effects of Tofa on the bone microenvironment. Studies on the impact of the other JAKi on bone marrow adiposity are needed to determine whether the stimulatory effect on bone marrow adiposity is a class effect or is specific to Tofa.

## Data Availability Statement

The original contributions presented in the study are included in the article/**Supplementary Material**. Further inquiries can be directed to the corresponding author.

## Ethics Statement

The studies involving human participants were reviewed and approved by the Institutional Review Board of CHU de Lille (2019–001159–37) and the National Human Experimentation Ethics Committees (reference CPP 40/19). The patients/participants provided their written informed consent to participate in this study.

## Author Contributions

J-GL and AC performed the experiments. J-GL, JP, and AC wrote the manuscript. All authors listed have made a substantial, direct, and intellectual contribution to the work and approved it for publication.

## Funding

JGL was supported by a PhD grant from Société Française de Rhumatologie and from ISite-ULNE. The TOFAT study received funding from Pfizer. The funder was not involved in the study design, collection, analysis, interpretation of data, the writing of this article or the decision to submit it for publication. All authors declare no other competing interests.

## Conflict of Interest

The authors declare that the research was conducted in the absence of any commercial or financial relationships that could be construed as a potential conflict of interest.

## Publisher’s Note

All claims expressed in this article are solely those of the authors and do not necessarily represent those of their affiliated organizations, or those of the publisher, the editors and the reviewers. Any product that may be evaluated in this article, or claim that may be made by its manufacturer, is not guaranteed or endorsed by the publisher.

## Supplementary Material

Supplementary Figure 1 Adipogenic differentiation of hBMSCs was confirmed **(A)** by using Q-PCR analysis of mRNA levels of adipogenic markers (PPARy, C/EBPα, PLIN 1) performed over a 14 days period of induction from day 0 (control hBMSCs) and Oil Red O staining. Osteogenic differentiation of hBMSCs was confirmed **(B)** by using Q-PCR analysis of mRNA levels of osteogenic markers (RUNX2, OC) performed over a 14 days period of induction from day 0 (control hBMSCs), ALP activity and Alizarin Red Staining at 21 days of differentiation. The data are representative of three independent experiments.

## Abbreviations

bDMARDs, Biological Disease-Modifying Antirheumatic Drugs; BMAds, Bone Marrow Adipocytes; BMD, Bone Mineral Adiposity; C/EBPα, CCAAT/enhancer-binding protein alpha; CRP, C Reactive Protein; csDMARDs, Conventional Synthetic Disease-Modifying Antirheumatic Drugs; CTX, C-terminal collagen crosslinks; Dlx5, Distal-less homeobox 5; DMEM, Dulbecco’s Modified Eagle Medium; DXA, Dual-energy X-ray Absorptiometry; FCS, Fetal Calf Serum; hBMSC, human bone marrow-derived stromal cell; IL, interleukin; IPAQ-SF, International Physical Activity Questionnaire—Short Form; JAK, Janus Kinase; JAKi, JAK inhibitors; MRI, Magnetic Resonance Imaging; Ob, Osteoblast; PBS, Phosphate Buffered Saline; PCR, Polymerase Chain Reaction; PDFF, Proton Density Fat Fraction; PLIN, Perilipin; PPARγ2, Peroxisome Proliferator-activated Receptor gamma 2; RA, Rheumatoid Arthritis; RNA, Ribonucleic Acid; Runx2, Runt-related transcription factor 2; STAT, Signal Transducer and Activator of Transcription; TNFα, Tumor Necrosis Factor alpha; Tofa, Tofacitinib; WST-1, Water Soluble Tetrazolium Salts-1; Ywhaz, tyrosine 3-monooxygenase/tryptophan 5-monooxygenase activation protein.
