# Supplementary figures and images for: Stimulatory Effect of Tofacitinib on Bone Marrow Adipocytes Differentiation
Source: Front Endocrinol (Lausanne). 2022 Jul 6;13:881699. doi: 10.3389/fendo.2022.881699 (PMC9299421; doi:10.3389/fendo.2022.881699)

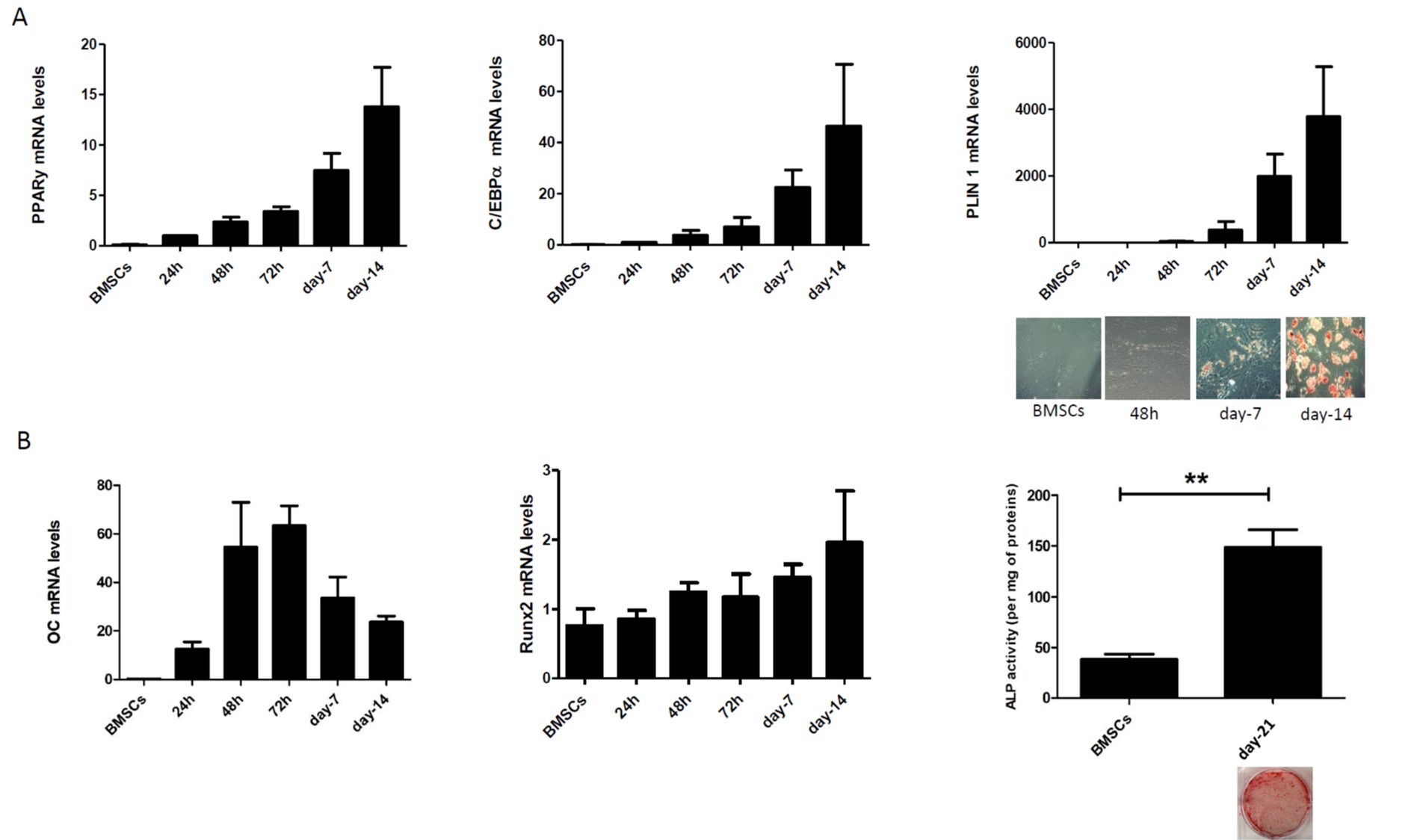

Supplement: Supplementary Figure 1 — Adipogenic differentiation of hBMSCs was confirmed (A) by using Q-PCR analysis of mRNA levels of adipogenic markers (PPARy, C/EBPα, PLIN 1) performed over a 14 days period of induction from day 0 (control hBMSCs) and Oil Red O staining. Osteogenic differentiation of hBMSCs was confirmed (B) by using Q-PCR analysis of mRNA levels of osteogenic markers (RUNX2, OC) performed over a 14 days period of induction from day 0 (control hBMSCs), ALP activity and Alizarin Red Staining at 21 days of differentiation. The data are representative of three independent experiments. [file Image_1.jpeg]
